# What Early Sapiens Cognition Can Teach Us: Untangling Cultural Influences on Human Cognition Across Time

**DOI:** 10.3389/fpsyg.2020.00099

**Published:** 2020-01-31

**Authors:** Andrea Bender

**Affiliations:** ^1^SFF Centre for Early Sapiens Behaviour (SapienCE), University of Bergen, Bergen, Norway; ^2^Department of Psychosocial Science, University of Bergen, Bergen, Norway

**Keywords:** cognition, culture, evolution, early humans, *chaîne opératoire*, cross-cultural comparisons, phylogenetic comparative methods

## Abstract

Evidence of cultural influences on cognition is accumulating, but untangling these cultural influences from one another or from non-cultural influences has remained a challenging task. As between-group differences are neither a *sufficient* nor a *necessary* indicator of cultural impact, cross-cultural comparisons in isolation are unable to furnish any cogent conclusions. This shortfall can be compensated by taking a diachronic perspective that focuses on the role of culture for the emergence and evolution of our cognitive abilities. Three strategies for reconstructing early human cognition are presented: the *chaîne opératoire* approach and its extension to brain-imaging studies, large-scale extrapolations, and phylogenetic comparative methods. While these strategies are reliant on our understanding of present-day cognition, they conversely also have the potential to advance this understanding in fundamental ways.

## Investigating the Role of Culture for Cognition

Human cognition is profoundly shaped by culture. This is perhaps more evident for some domains than for others, but striking examples abound: orientation in and referencing to space and time (Levinson, 2003; Haun et al., 2011; Bender and Beller, 2014b), reasoning about the biological world (Medin and Atran, 2004; Bang et al., 2007; Ojalehto et al., 2017a, b), accounting for cause–effect relations (Choi et al., 1999; Bender and Beller, 2019), or representations of numbers (Beller and Bender, 2008; Bender and Beller, 2012, 2014a; Núñez, 2017), not to mention the way in which we conceptualize social relationships (Fiske, 1992; Lillard, 1998) and ourselves within them (Markus and Kitayama, 1991). As much as they are rendered possible by endowed capacities, all of these cognitive abilities, activities, and achievements are also predicated on culture, be it by way of culturally accumulated and transmitted knowledge, culture-specific concepts and framework theories, cultural tools, conventions, and practices, or simply by the fact that we are a cultural species (Tomasello et al., 2005; Henrich, 2016; Heyes, 2018; Bender, 2019).

### Limitations of Cross-Cultural Studies

Despite increasing evidence for the existence of cultural influences on cognition, untangling them from one another or from non-cultural influences has remained a challenging task. One obvious, and increasingly popular, strategy is cross-cultural comparison. Such studies do help us to detect diversity in cognitive phenomena, but they do not reveal the extent to which this diversity is brought about by culture. For instance, ethnolinguistic groups differ regarding which frame of reference they habitually adopt for representing spatial relations. Whether, however, these differences are due to linguistic availabilities, to cultural preferences, or to environmental affordances and constraints is subject to ongoing debate (overviews in Majid et al., 2004; Bohnemeyer et al., 2014).

While such differences are interesting in any case, in that they attest to cognitive diversity, for us to be willing to accept them as *cultural* differences, we would want them to involve at least two types of social processes: one for generating the pattern (such as transmission) and one for stabilizing it (such as mutual coercion). After all, the hallmarks of culture are learning, sharing, and some extent of normativity (Brumann, 1999; Gatewood, 2012; de Munck and Bennardo, 2019). As a minimum standard, therefore, cross-cultural studies would have to be complemented and bolstered by in-depth ethnographic investigations of where these group-specific patterns originate, how they spread, and how they are maintained (for a rare example of this combination, see Dasen and Mishra, 2010). Another strategy for teasing apart influences of culture from other factors is triangulation: comparing one group with two others that differ from the former on distinct dimensions, one regarding culture, the other regarding, say, environmental experience (Medin and Atran, 2004).

In addition to not being *sufficient* as indicators of cultural influences, between-group differences are not *necessary* either. Theory of mind, for instance, is the ability for mental reasoning, which humans typically acquire long before adulthood. It may thus seem to be a textbook example of an ability with which humans are endowed. Still, its development benefits from, if not relies on, social interaction and cultural practices, including a focus on mental states and the use of mentalistic language (Ruffman et al., 2002; Pyers and Senghas, 2009; overview in Träuble et al., 2013; Slaughter and Perez-Zapata, 2014). As a consequence of culture’s pivotal role in the development of this ability, all human populations – unlike any other species – become so attuned to reasoning about others’ minds that they cannot help but also ascribe some form of mental reasoning to species that, for all we know, most likely lack this ability (Povinelli and Vonk, 2003). That is, culture impacts on cognition not only as a means of diversification, but also, and profoundly so, as a driving force in cognitive evolution and development. Yet, if asserting whether between-group differences in cognitive behavior are caused by culture is already challenging, then asserting an influence of culture in those instances in which it does not even produce any differences poses seemingly insurmountable obstacles.

In short, cross-cultural comparisons are a valuable scientific tool for the investigation of cognitive diversification, but in isolation they fall short of furnishing any cogent conclusions: While they allow us to detect differences, they do not allow us to infer an impact of culture from the presence of such differences, or indeed to infer a lack of impact from their absence. Crucially, cross-cultural comparisons remain largely silent on the scaffolding role of culture – a shortfall that can be compensated by adopting an evolutionary perspective on how aspects of specifically human cognition emerged.

### The Potential of an Evolutionary Perspective

Assessing the role of culture for early human cognition is important both on ontological and epistemic grounds: It is a subject worthy of investigation in its own right, but it is also an essential qualification in all attempts to reconstruct past cognition.

Several hundred thousand years ago, early *Homo sapiens* learned to control fire, invented complex tools such as bow-and-arrow sets, and began to use abstract symbols and language (Henshilwood et al., 2002, 2018; Wadley, 2013; Backwell et al., 2018). Even in hindsight, these achievements strike us as truly impressive, yet what made them possible has remained one of the most tantalizing questions of human evolution. Among paleoscientists, there is increasing consensus that most, if not all, important innovations made by *Homo sapiens* were brought about by evolutionary mechanisms in which culture was the major driving force (for more details and examples, see Colagè and d’Errico, 2018; Bender, 2019; Sterelny, 2019). One such mechanism is the cultural transmission and accumulation of knowledge, ideas, and inventions, which generates the “ratchet effect” so characteristic of human culture (Tennie et al., 2009; Henrich, 2016). It is attested to, for instance, in numerical cognition, in which a concept, once understood, paves the way for further elaboration (Miller and Paredes, 1996). Existing cultural achievements may also be co-opted for new cognitive purposes, a mechanism that is called cultural exaptation and has been detailed for number notations (d’Errico et al., 2017; d’Errico and Colagè, 2018). Even brain anatomy and gene pool are impacted by culture, through mechanisms such as cultural neural reuse and gene-culture coevolution: the former by recycling cortical maps, as in the case of literacy (Dehaene and Cohen, 2007; d’Errico and Colagè, 2018), the latter by exerting pressure on gene selection (Boyd and Richerson, 1988; Laland et al., 2010). Our language abilities, for instance, are linked to genetic mutations that are present in the human line, but not in other primates. While, according to a standard scenario of pure natural selection, language use would have been “switched on” in individuals possessing the mutation, gene-culture co-evolution opens up a more convincing, alternative scenario: that some form of language use was already part of the cultural environment, in which the mutation could then confer a selective advantage (Fisher and Marcus, 2006; Fisher and Ridley, 2013).

In short, culture helped us to develop cognitive capacities (such as language), cognitive tools (such as writing), and cognitive skills (such as calculation). Investigating the mechanisms that sparked off and shaped these capacities, tools, and skills is therefore essential, as it will enable us to illuminate the instrumental role of culture.

## Reconstructing Early Human Cognition

When trying to delineate the impact of culture on the emergence, evolution, and molding of what makes human cognition unique, we face a major challenge. Other than the material remains of humans and the material output of their activities, the cognitive skill set and knowledge available to our ancestors have left no direct traces in the archeological record and therefore have to be inferred. Three such approaches from a wide range of disciplines are detailed below: reconstructing the cognitive underpinnings of past activities, large-scale extrapolation of cognitive abilities, and retracing the cultural evolution of cognitive systems.

### Reconstructing Cognition Involved in “Past Activities”

The approach generally adopted in cognitive archeology is a kind of reverse engineering. Taking the material remains of an artifact as a starting point, the implicated *chaîne opératoire* is then deployed to reconstruct the steps required for its production, ranging from acquisition of the raw material to manufacturing and subsequent use. It then goes from the behavioral components involved in these processing steps back further to infer the cognitive and social endowment indispensable for production, such as working memory capacity or focus of attention (Sellet, 1993; Haidle, 2010, 2014).

Lombard and Haidle (2012) elaborated this type of analysis for the production of bow-and-arrow sets, which date back to at least the Middle Stone Age in Sub-Saharan Africa (Backwell et al., 2018). This complementary tool set, in which the extraordinary efficiency of the components only unfolds when they are used jointly, was a major technological advancement, linked to an increase in cognitive and behavioral flexibility. Its invention required the craftsperson to conceive of a novel idea, for which several unrelated elements needed to be detached from their distinct context and combined to form something entirely new. This ability to assemble objects and actions in a modular manner is considered as a major breakthrough in problem-solving and creativity (Lombard and Haidle, 2012).

Such reconstructions of the cognitive, behavioral, and social components necessary for the production of prehistoric tools are increasingly complemented by neuroscientific methods (Stout and Chaminade, 2007). To identify the neural substrates involved in past activities such as flint-knapping, the brain activation of present-day participants is measured while they engage in mental imagery of these activities. This helped researchers to ascertain, for instance, that more advanced tool-making requires more efficient visuomotor coordination and hierarchical action organization, and points to a shared basis of tool-making and language (Stout et al., 2008). More recently, Mellet et al. (2019) adopted a similar strategy to investigate symbol processing. They found that prehistoric engravings are perceived by present-day participants as organized and meaningful representations, which suggests a symbolic function also for those who created them.

These reconstructive approaches all share the assumption that our human ancestors possessed an almost identical genome, similar brain structures, and hence basically the same cognitive capacities as do contemporary humans. While this assumption is plausible, the inference that early sapiens cognition can therefore be simply extrapolated from today’s cognition is more contestable. This inference is valid only to the extent that it takes into account the changes brought about by cultural evolution and ensuing cognitive diversification.

### Reconstructing “Past Cognition” by Extrapolation

When attempting to extrapolate from present-day cognition to past cognition, two major challenges need to be tackled: One is to delineate basic aspects from all of those that are generated by mechanisms of cultural evolution, and the other is to delineate universal aspects from all of those that are shaped by culture. These two tasks require different approaches.

The delineation of *basic* aspects of cognition is aided by insights from a wide range of disciplines including evolutionary anthropology and paleoanthropology, archeology, comparative psychology, and language evolution. This research helps to identify the set of social and cognitive skills that is uniquely human (Haun et al., 2010; Tomasello and Herrmann, 2010), the evolutionary mechanisms that enabled them, such as cumulative culture and cultural exaptation (Tennie et al., 2009; Colagè and d’Errico, 2018; Heyes, 2018), and characteristics of those processes and constraints that continue to shape cognitive tools and skills (Christiansen and Chater, 2008; Lupyan and Dale, 2016). The classic example of a cultural innovation with a profound impact on cognition is literacy. Learning to read and write not only facilitates the accumulation and transmission of knowledge on a grand new scale (Huettig and Mishra, 2014; Morin et al., 2018), but also rewires the individual brain (Dehaene and Cohen, 2007). When extrapolating to past cognition, we therefore need to discount the neural and cognitive changes brought about by cultural innovations such as literacy and, more generally, we need a more exhaustive overview of the range of changes to be taken into account.

For delineating *universal* aspects of cognition, large-scale cross-cultural studies are an important step (e.g., Majid et al., 2004, 2015; Henrich et al., 2005, 2010a; Bohnemeyer et al., 2014). For the case of language, such studies have found substantial diversity on almost every level of linguistic organization (Evans and Levinson, 2009; Dunn et al., 2011). The universals that could be established so far seem to be confined to aspects of usage such as turn-taking and repair (Dingemanse et al., 2015; Levinson, 2016). A better understanding of what is really shared by present-day humans, however, is a prerequisite for any attempt to extrapolate our models and assumptions to early sapiens cognition.

Yet, as noted before, the identification of diversity can only be the first in a series of steps, and needs to be enriched by deep ethnographic understanding. When combining cross-cultural studies with investigations into cognitive development and across species (Liebal and Haun, 2018), we are able to more accurately assess the proportions of cultural diversity and convergence that are due to hereditary predispositions compared to those transformed by cultural influences (Haun et al., 2006). An even more comprehensive combination of approaches was tested recently for causal cognition (Bender and Beller, 2019): Research across species, back into prehistory, and on cognitive development was surveyed to identify those aspects of causal cognition that are specific to humans rather than shared by other primates, and research across cultures and languages was surveyed to identify both commonalities and differences. One specifically human feature is the pivotal role of causally relevant knowledge, most of which is culturally accumulated and transmitted. As a consequence, causal cognition in humans is shaped by culture in two ways: It is diverse across cultural traditions, and it is molded more generally by the distinct characteristics of human sociality, modes of teaching, and language.

### Reconstructing “Past Cognition” From Present Diversity

While the previously mentioned strategies try to take cultural evolution and diversity into account on inferential grounds, the third approach, originating from evolutionary biology and anthropology, capitalizes precisely on present-day diversity, as reflected, for instance, in conceptual systems. In principle, diversity in such systems is understood to be an outcome of evolutionary processes, either due to random changes or to systematic transformations. With the help of phylogenetic comparative methods, evolution can therefore be “re-run” in order to gauge the relative proportions of these sources. The same approach also allows one to infer past states of such systems, to assess their transformations, and to retrace co-evolution between system properties and other factors (Mace and Holden, 2005; Levinson and Gray, 2012).

Let us illustrate its explanatory power for systems of kinship terminology (Jordan, 2013). Given the clear and strong biological underpinnings of kin relations, the degree of diversity exhibited by kinship systems is astonishing – and still by no means unlimited (Levinson, 2012). Differences occur particularly in the extent to which features such as generation, gender, relative age, or connecting relatives are relevant for classification. Some systems, for instance, collate cousins with brothers and sisters, while others distinguish between same- and opposite-sex cousins of same- or opposite -sex parents (rendering some of them prohibited and some desired marriage partners). Harnessing phylogenetic comparative methods has helped to identify the semantic distinctions made in a kinship system, or the settlement patterns based thereupon, in ancestral populations and to assess how these have changed over time (Jordan et al., 2009; Jordan, 2011). The same methods can also be deployed to test hypotheses regarding cognitive constraints on a system’s complexity. For the case of kinship systems, several constraining factors have been identified, among them general communicative principles that balance informativeness and simplicity, language-specifics such as descent from a joint ancestor language, and the social practices linked to the kinship systems (Kemp and Regier, 2012; Rácz et al., 2019).

In a nutshell, the modeling of evolutionary trajectories enables us to ascertain the primal states of cognitive systems and to reconstruct the conditions under which such states are likely to change. This renders it a powerful tool for reconstructing past cognition from present-day diversity, specifically in those domains for which rich phylogenetic language trees are available.

## Conclusion

The strategies presented here are an important step toward the reconstruction of early sapiens cognition, but their true potential is greater. While each of these strategies is reliant on our understanding of present-day cognition, they also substantially advance this understanding, including an appreciation of how cognition is influenced by culture. More concretely, they help to delineate those aspects of cognition that are widely shared and universal, and allow us to assess more general constraints on diversity. They help to illuminate the conditions under which aspects of cognition emerged, evolved, and changed, as well as the time scales in which this happened. By retracing evolutionary trajectories, they help to identify components with which a cognitive skill or tool may have co-evolved. And by identifying these driving forces in cognitive evolution, finally, they also raise our awareness of the extent to which these factors continue to impact on cognition.

Since its emergence, cognitive science has been strongly committed to the assumption that cognition basically works in the same way across all human populations (Flanagan, 1991) – a view still popular in wide parts of cognitive psychology. The insight that variability in cognition may indeed be greater than long assumed is slowly gaining ground (Bender et al., 2015; Bender, 2019). An accumulation of empirical findings (e.g., Levinson, 2003; Medin and Atran, 2004; Bender and Beller, 2016) and methodological criticism from within the cognitive sciences (Arnett, 2008; Henrich et al., 2010b) has helped to promote an attitude change. But the research field still has a long way to go from acknowledging the existence of cognitive diversity to accepting the profound impacts of culture, or to be able to untangle the processes involved. The position championed here is that real progress in this regard will depend on combining different perspectives in an interdisciplinary effort to investigate cognition across both cultures and time. We have a lot to learn from today’s cognitive diversity for the evolution of human cognition, but we have equally much to learn from our ancestors for today’s cognition.

## Author Contributions

The author confirms being the sole contributor of this work and has approved it for publication.

## Conflict of Interest

The author declares that the research was conducted in the absence of any commercial or financial relationships that could be construed as a potential conflict of interest.
